# Causal analysis and visualization of magnetization reversal using feature extended landau free energy

**DOI:** 10.1038/s41598-022-21971-1

**Published:** 2022-11-29

**Authors:** Sotaro Kunii, Ken Masuzawa, Alexandre Lira Fogiatto, Chiharu Mitsumata, Masato Kotsugi

**Affiliations:** 1 Faculty of Advanced Engineering, Tokyo University of Science, Tokyo, 125-8585 Japan; 2 National Institute for Materials Science (NIMS), Tsukuba, 305-0047 Japan

**Keywords:** Theory and computation, Ferromagnetism, Magnetic properties and materials, Characterization and analytical techniques, Ferromagnetism, Magnetic properties and materials

## Abstract

The magnetization reversal in nanomagnets is causally analyzed using an extended Landau free-energy model. This model draws an energy landscape in the information space using physics-based features. Thus, the origin of the magnetic effect in macroscopic pinning phenomena can be identified. The microscopic magnetic domain beyond the hierarchy can be explained using energy gradient analysis and its decomposition. Structural features from the magnetic domains are extracted using persistent homology. Extended energy is visualized using ridge regression, principal component analysis, and Hadamard products. We found that the demagnetization energy concentration near a defect causes the demagnetization effect, which quantitatively dominates the pinning phenomenon. The exchange energy inhibits pinning, promotes saturation, and shows slight interactions with the defect. Furthermore, the energy distributions are visualized in real space. Left-position defects reduce the energy barrier and are useful for the topological inverse design of recording devices.

## Introduction

The magnetization reversal phenomenon is an important function as it determines the information-recording ability of spintronic devices and driving efficiency of motors^1–3^. Macroscopic magnetization reversal is characterized by changes in the microscopic magnetic domain. A magnetic domain structure is constructed by a complex interaction depending on the demagnetization, exchange energies, and the location of defects. These aspects change the energy barrier required for magnetization reversal, resulting in domain wall pinning^4–6^. The energy required for magnetization reversal is directly related to the recording power-consumption in spintronic devices and coercivity in permanent magnets. Microfabrication of magnetic discs has been performed in spintronics devices to reduce the reversal energy cost by controlling pinning strength^5–10^. Generation of a magnetic vortex is controlled by the size and interface of the magnetic disc^11,12^. The coercivity of permanent magnets is controlled by precipitating a nonmagnetic material at the interface^9^. Fundamentally, spatially inhomogeneous systems with discontinuous boundaries exhibit various attractive physical properties, such as topological defects^13,14^, maze magnetic domains^15^, skyrmions^14^, magneto-ionic heterostructures^16^, and antiferromagnetic frustrated systems^13^. Structure-to-property links have been a longstanding issue in mesoscale physics^13–16^. Local magnetic domains have been analyzed based on qualitative visual analysis^10–15^. A few attempts have been made towards quantitative structure-to-property link and causal analyses using material bigdata. Thus, the interconnection of microscopic magnetic domains and macroscopic reversal phenomena is an important open issue involving not only applied devices but also mesoscale physics.

Pinning phenomena in permalloy (Py) nanomagnets are an important example of spintronic devices^17^. Intentionally designing microstructures, such as defects and notches, has been a traditional strategy to improve the reversal field^18–20^. Pinning phenomena manifest as the Barkhausen jump^5,18^, which is a series of sudden changes in the size and orientation of magnetic domains or microscopic clusters of aligned atomic magnets and occurs during the reversal process. It has been qualitatively described by complex changes in magnetic domains due to multiple interactions between defects, demagnetization energy of the long-range effect, and exchange energy of the short-range effect induced by an external magnetic field. Pinning is strongly influenced by the defect location. Py dots with centered defects are symmetric and simple to analyze. Shifting the defects off-center changes the local equilibrium magnetization configurations. It alters the reversal process, leading to a large variation in Barkhausen jump^20^. In such inhomogeneous systems, it is extremely difficult to establish the relationship between the microscopic magnetic domain and macroscopic reversal process^10^. Moreover, analytical methods to identify the cause of complex interactions do not exist. Thus, a methodology for analyzing the pinning mechanism as well as the interactions, their locations, and their contributions to the macro functions is desirable.

**Figure 1:**
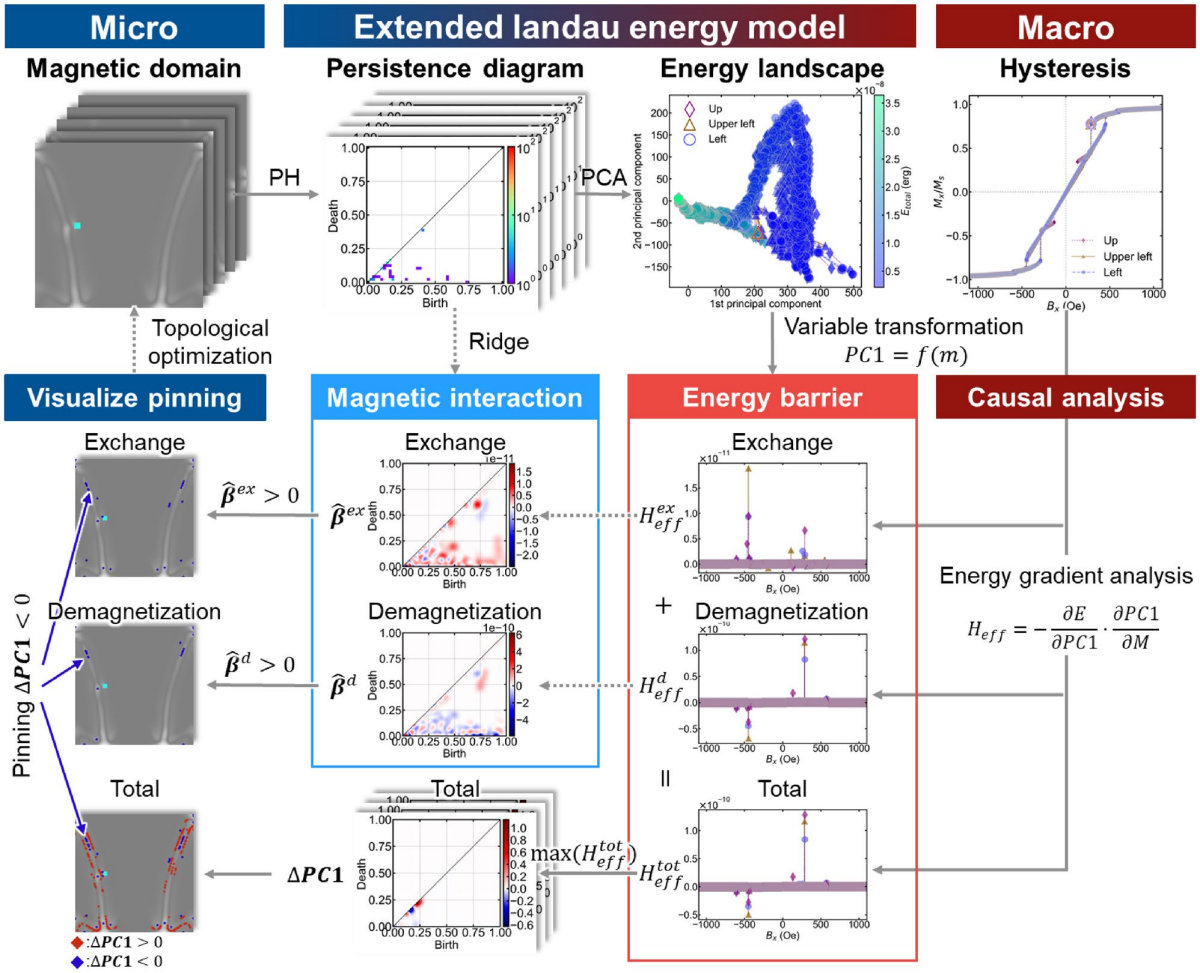
Workflow of magnetization reversal process analysis using the extended Landau free-energy model. PH is applied to the magnetic domain data to extract microstructural features (domain walls, magnetic vortices, etc.). PD and magnetic energy are correlated using Ridge regression. Principal Component Analysis is used to reduce the PD data to low dimensional space. An energy landscape is drawn on the information space by marking magnetic energy on each point. Latent variables obtained by PCA are features explaining both microscopic domain structure changes and macroscopic magnetization reversal process. They can be bidirectionally connected to various physical parameters. Detailed gradient analysis of the energy landscape is performed to extract the energy barriers dominating the pinning. A causal analysis of pinning is performed by decomposing the energy into each magnetic interaction and analyzing each magnitude. Finally, the dominating factors for pinning are visualized in the original magnetic domain while using Hadamard product.

An "extended Landau free-energy model" is designed by applying information science to the traditional Landau free-energy model (Fig. 1). This model effectively utilizes physics-based features to draw an energy landscape in the information space^21–23^. Discontinuous boundaries such as branching structures, end points, and vortex cores in magnetic domains can be described as topological defects^14^. The complexity of the magnetic domain containing topological defects was quantified using persistent homology (PH)^24–26^. PH is a powerful topological concept to quantitatively describe the structural features (size, shape, fluctuation, and connectivity) of holes and islands. PH is useful for describing medium-range information, which is difficult to detect via simple Fourier transformation, and is valuable for describing fine structure in magnetic domain^27^. PH can be combined with various machine learning techniques, and the obtained features are used as explanatory variables of energy landscapes.

The advantage of this model is that it analyzes the magnetization reversal process using physics-based features, such as energy and microstructure. By analyzing the correlation between the features and the various energy terms, the energy cost of structural change can be determined. The relationship between features and energy required for reversal can be established using variable transformation and first-order derivatives^22,23^. Moreover, magnetic energy can be linearly decomposed into exchange energy and demagnetization energy to quantitatively evaluate the origin of the magnetic effect. Hence, the use of physics-based features offers a highly explainable AI model for the analysis of the magnetic reversal phenomena, which is difficult to achieve with conventional deep learning^28^. Furthermore, the energy cost correlates with the macroscopic property in the system, and eigenvectors can be visualized back to the original magnetic domain structure, establishing a structure–property link. Bidirectional information transformation can connect the function and microstructure, enabling the design of a device structure to improve pinning^26^. Therefore, this method can be used in causal analysis, super-hierarchical analysis, and inverse design. This model is being systematically developed. A mathematical framework for feature extended energy modeling and its application to experimental data is reported^21,22^. The classical energy landscape was also discussed^21,29^.

Figure 1 shows the workflow of the causal and bidirectional analyses using the extended Landau free-energy model. We demonstrated an automated analysis of the magnetic effect governing the pinning phenomena in magnetization reversal process. The governing factor were visualized in the initial magnetic domain for inverse-designing device structures. First, we generated a magnetic domain dataset using micromagnetic simulations based on the Landau–Lifshitz–Gilbert (LLG) equation. We calculated the magnetic domains for various external fields and defect positions exhibiting the Barkhausen effect. Three types of off-center defect locations were set as model specimens: Up, Upper-left, and Left. By symmetry, Down and Down-left can be excluded. Coordinate dependence is not discussed here to develop the principles of the model. Second, we performed PH to extract the complexity of the magnetic domain as persistence diagrams (PDs). We performed ridge regression (RR) to link PD and magnetic energies. RR is a useful method to estimate the coefficients of multiple-regression model, in which independent variables are strongly correlated. A PD describes the complex spatial inhomogeneity of magnetization in the magnetic domain, such as the Bloch wall, magnetic vortices, and the fine structure inside the domain wall^26^. RR was performed with PD as the explanatory variable and magnetic energy as the objective variable. Bidirectional and quantitative linkages of the magnetic domain and energy were constructed using RR^30^. Third, we applied principal component analysis (PCA) to the PD to describe the trends of structural change. PCA determines the eigenvector explaining the most variation in the data as the first principal component. Energy landscapes were drawn in the information space using the latent variables obtained^30^. The PCA can represent complex data changes with fewer eigenvectors. Therefore, latent variables can explain the magnetization reversal process. The latent variable and energy are taken as the horizontal and vertical axes, respectively, to draw the energy landscape. Fourthly, we analyzed the local gradient of the energy landscape and identified the energy barriers for pinning. The total energy is decomposed into demagnetization and exchange energies. The governing energy term originating from magnetic interaction can thus be identified to understand the causal relationship. In the fifth step, we visualized the energy concentration that caused pinning in the original magnetic domain. Using the Hadamard product and logical sum, we extracted the dominant factors of pinning while maintaining the dimensions of the PD^31^. We visualized the cause of the magnetic effect onto the original magnetic domain image. An inverse design of the device structure was proposed.

## Results and discussion

### Magnetic domains and magnetization reversal processes using LLG simulations

**Figure 2:**
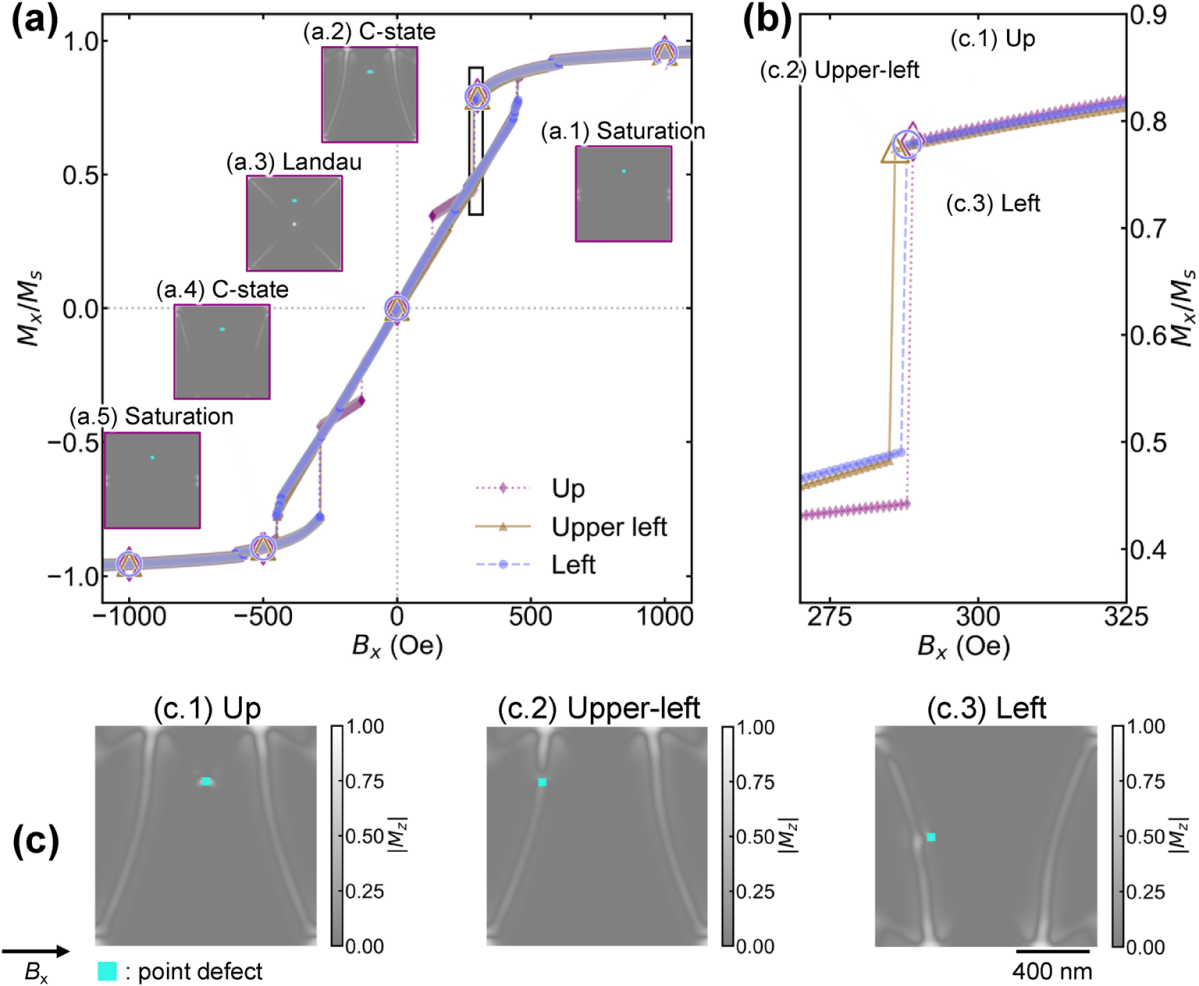
(**a**) Magnetic hysteresis and magnetic domain. The horizontal axis represents the external field *B*_x_ (Oe), and the vertical axis represents the normalized magnetization *M*_x_. Magnetic hysteresis is shown in the range of 1000 Oe to − 1000 Oe. Typical magnetic domains are shown as (**a.1**) Saturation, (**a.2**) C-state, (**a.3**) Landau, (**a.4**) C-state, and (**a.5**) Saturation, respectively. (**b**) Magnified inset of (**a**). For simplicity, only the reducing-field process is shown. Pinning and depinning cause a Barkhausen jump. Depending on the defect position, the depinning behavior changes slightly. (**c**) Magnetic domain in a pinning C-state. The microstructure of the magnetic domain differs slightly depending on the position of the defect (**c.1**) Up, (**c.2**) Upper-left, and (**c.3**) Left. The defects are indicated in light blue.

Figure 2a shows the magnetic hysteresis and the corresponding magnetic domains simulated by LLG calculation. Representative magnetic domains for the Up defect are shown in Fig. 2a.1–a.5. We qualitatively described the magnitude of the Barkhausen jump for each system in a classical manner. The magnetization of the Py dots was saturated at an external field of 1000 Oe, exhibiting a single magnetic domain (a.1). A magnetic domain called the C-state was constructed at approximately 400 Oe (a.2). The C-state is divided into a wide magnetic domain in the central area and reversed magnetic domains on the left and right sides. A flux closure vortex magnetic domain constructed near 0 Oe is called “Landau pattern” (a.3)^32,33^. The C-state magnetic domain was constructed again near 500 Oe (a.4). The vortex core was pushed out to construct the C-state during magnetization reversal. The magnetic dots saturate in the reverse direction at − 1000 Oe (a.5). The dataset of the magnetic domain was prepared for magnetic dots with Upper-left and Left defect positions (Supplemental Fig. 1). The simulation results accurately reproduced the experimental magnetic domain.

Figure 2b shows a magnified view of Fig. 2a near 300 Oe. The magnetization abruptly decreases. The magnetic domain of the C-state shows pinning and depinning, resulting in the Barkhausen jump^5,33^. The magnitude of the Barkhausen jump depends on the defect location, with the Up (c.1) dot being the largest, followed by the Upper-left (c.2) and Left (c.3) dots. An earlier study reported that there is an energy barrier from the C-state to the stable Landau pattern^34^. When the system gains Zeeman energy from the external field, the internal magnetic domain changes in a complex manner and overcomes the barrier^35–37^. However, it is difficult to decompose the complex interactions from visual analysis.

Figure 2c shows the magnetic domains of the C-state for Up, Upper-left, and Left, shown as (c.1), (c.2), and (c.3), respectively. Focusing on the location, in Up, the defects were sufficiently far from the domain wall. However, in Upper-left (c.2) and Left (c.3), the defects and domain walls are sufficiently close to each other, possibly because of an interaction. Figure 2b also shows that the Barkhausen jump of Up (c.1) is larger than those of Upper-left (c.2) and Left (c.3). We can assume an interaction between the domain wall and the defects may influence the magnitude of the Barkhausen jump.

### Ridge regression from persistence diagram to demagnetization energy and exchange energy

The energy barrier of pinning is defined as the total energy difference over the Barkhausen jump. Correlating the energy and magnetic domains could be the key to understanding the pinning mechanism^35^. The total energy is the sum of the exchange energy from the exchange interaction and demagnetization energy from the dipole interaction. The complexity of the magnetic domain is governed by a delicate balance between the two magnetic energy terms^32^. In a previous study, the PH was used to quantify the complexity of the magnetic domain. Bloch walls, magnetic vortices, and fine structures of the domain walls were characterized as PD^26^. Although both simulated and predicted energy can be used for landscape analysis, it is useful to confirm the prediction accuracy and the availability of the features. Here, we construct the relationship between PD and energy by RR. The advantage of RR is that the analytical solution can be obtained by L2 regularization, which avoids the problem of multicollinearity and allows for accuracy while improving generalization performance. The mathematical formula is

1 $$ \hat{\user2{\beta }} = \left( {{\varvec{X}}^{T} {\varvec{X}} + k{\varvec{I}}_{{\varvec{p}}} } \right)^{ - 1} {\varvec{X}}^{T} {\varvec{y}}, $$

where $\hat{\user2{\beta }}$ is the regression coefficient, ${\varvec{X}}$ is the input vector data of the PDs generated from the magnetization reversal processes of the three samples, *k* is the regularization parameter, and ${\varvec{I}}_{{\varvec{p}}}$ is a unit vector. The objective variables were set to each energy term, and the explanatory variables were matrices generated from PDs^30^. We evaluated the prediction accuracy with the coefficient of determination obtained and determined the regression coefficient vector $\hat{\user2{\beta }}$.

**Figure 3:**
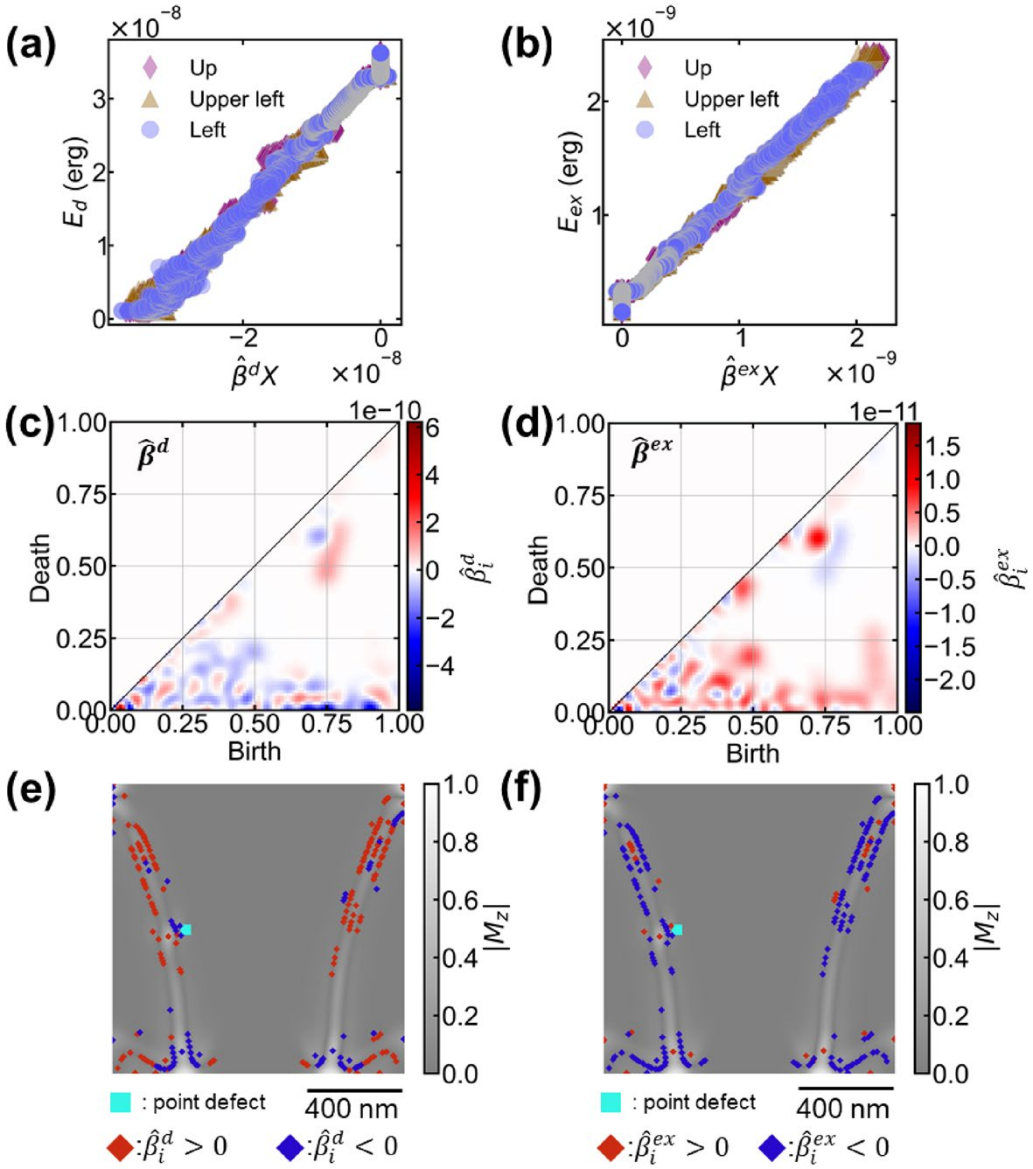
Relationship between magnetic energies ($E_{d}$ and $E_{ex}$) and regression coefficients $\hat{\user2{\beta }}$ ($\hat{\user2{\beta }}^{d}$ and $\hat{\user2{\beta }}^{ex}$) using RR. Visualization results to the original magnetic domain. (**a**) Relationship between the demagnetization energy and obtained score $\hat{\user2{\beta }}^{d} \cdot {\varvec{X}}$. The horizontal axis is the inner product of the regression coefficient and PD ($\user2{\hat{\beta }X}$), and the vertical axis is the demagnetization energy. ${\varvec{X}}$ is the input vector data in the PD of one loop of magnetic hysteresis. The determination coefficient obtained is $R^{2}$ = 0.995. (**b**) Relationship between the exchange energy and the obtained score $\hat{\user2{\beta }}^{ex} \cdot {\varvec{X}}$. The horizontal axis is the inner product of the regression coefficient and PD ($\user2{\hat{\beta }X}$), and the vertical axis is the exchange energy. The determination coefficient is $R^{2}$ = 0.993. (**c**) and (**d**) are reconstructed PDs from the regression coefficients $\hat{\user2{\beta }}^{d}$ of the demagnetization energy and $\hat{\user2{\beta }}^{ex}$ of the exchange energy, respectively. The blue-red color scale indicates the weights for ${\varvec{X}}$ in calculating each energy. (**e**) and (**f**) are the visualization of the regression coefficients of the demagnetization and exchange energies to the magnetic domain, respectively. Points visualized from positive elements ($\hat{\user2{\beta }}_{d} > 0$) are shown in red and negative elements ($\hat{\user2{\beta }}_{d} < 0$) are shown in blue.

Figure 3a,b show the energy analysis using the RR. $\hat{\user2{\beta }}$ is simply obtained by RR. $\user2{\hat{\beta }X}$ and each energy term exhibit a high correlation in both (a) and (b). The coefficients of determination R^2^ are (a) R^2^ = 0.995 for the demagnetization energy and (b) R^2^ = 0.993 for the exchange energy. RR could predict the exact energies calculated with Mumax3 with more than 99% accuracy. This allows us to correlate the regression coefficient $\hat{\user2{\beta }}$ to each energy term. $\hat{\user2{\beta }}$ could be a useful feature to explain the demagnetization and exchange energies. $\hat{\user2{\beta }}$ is the regression coefficient obtained by L2 regularization. $\hat{\user2{\beta }}$ maintains the same dimensions of PD (Fig. 3c,d). Figure 3c,d show the PD contributing to the demagnetization and exchange energies, respectively. RR constructed the relationship between energy and $\hat{\user2{\beta }}$. Positive values in the regression coefficient vector $\hat{\user2{\beta }}$ indicate a positive correlation with the target variable (energy), while negative values indicate a negative correlation. The components of $\hat{\user2{\beta }}$ are positively and negatively inverted for demagnetization energy and exchange energy (Fig. 3c,d). Generally, magnetic domains are formed by balancing the energy by increasing the exchange energy to decrease the demagnetization energy. The RR results are self-consistent with physical phenomena. $\hat{\user2{\beta }}$ is a vector describing the energy transfer in the magnetic domain structure change.

We visualized the contribution of each energy term back to the initial magnetic domain structure using $\hat{\user2{\beta }}$^26^. Figure 3e shows the visualization results of the contributing factor of the demagnetization energy $\hat{\user2{\beta }}_{d}$ in the magnetic domain. The results for the Left defect are presented as an example. The coordinates for $\hat{\user2{\beta }}_{d} < 0$ are distributed around the defect and at the edge of the dot. Conventionally, magnetic poles are produced at the edges and accompanied by an increasing demagnetization energy. $\hat{\user2{\beta }}_{d} < 0$ contributes to increase the demagnetization energy. Hence, it may detect the magnetic poles produced at the edges and accompanying demagnetization-energy increase, which is a reasonable result. Notably, only the coordinates can be visualized, and the original image cannot be reconstructed. The coordinates of $\hat{\user2{\beta }}_{d} > 0$ are distributed at the end of the wide magnetic domain, i.e., on both sides of the magnetic wall. Many generators were produced at the PD sublevels, which is a filtration technique extracting weak fine structures from the image (Supplemental Fig. 2). It visualized the coordinates where the *M*_z_ component began to appear (Supplemental Fig. 3). Thus, we can visualize the spatial distribution of demagnetization energy. Demagnetization can inhibit magnetic wall propagation. Therefore, it is useful for understanding magnetization reversal.

Figure 3f shows a visualization of the contributors of the exchange energy using $\hat{\user2{\beta }}_{ex}$. The coordinates $\hat{\user2{\beta }}_{ex} > 0$ are distributed in the center of the domain wall, where the exchange energy increases. It is a reasonable result because the center of the domain wall is the location where the exchange energy increases due to twisting of magnetization. The coordinates of $\hat{\user2{\beta }}_{ex} < 0$ were distributed on both sides of the domain wall. Many generators also appear at the sublevels in this PD (Supplemental Fig. 2). It visualized the coordinates where the rotation of magnetization is lost at the side of the domain wall. The visualized coordinates are almost identical in terms of demagnetization energy and exchange energy, but the signs are reversed. The magnetization rotation loss corresponds to where the *M*_z_ component begins to appear. It indicates that the demagnetization energy and exchange energy act at approximately the same location, with opposing contributions. In contrast to the traditional explanation of the magnetic domain, the shape of the domain wall is determined by the balance between demagnetization energy and exchange energy. This result illustrates the bidirectional relationship between the magnetic domain and each magnetic energy term. It should be an interesting visualization to be able to trace individual magnetic energy contributions back to the original magnetic domain. A detailed causal analysis of the pinning is described in section “Visualization of causal magnetic effects on the initial magnetic domain”.

### Drawing energy landscapes using extended Landau model

We used extended Landau free energy to analyze energy changes and feature distances and interpret the magnetization reversal process. The extended Landau free-energy model draws the energy landscape of the system in the information space with features as explanatory variables. As in conventional Landau theory, the magnetization reversal process traces a minimal path in the energy landscape. The magnetic domain changes continuously with the external field. Its features are extracted as the PD. The explanatory variables for the extended Landau free-energy model were obtained by principal component analysis. The features were latent variables that explained the magnetization reversal process well. The total energy of the magnetic domain is the sum of the demagnetization and exchange energies. The magnetic domain is constructed to minimize the total energy. By RR, each magnetic energy and PD allows bidirectional information transformation. The PD is a vector explaining the energy change with the same dimension as the eigenvector. In other words, the inner product between the PD and eigenvector describes the magnetic domains. It is useful for analyzing the energy landscape in the magnetization reversal process.

**Figure 4:**
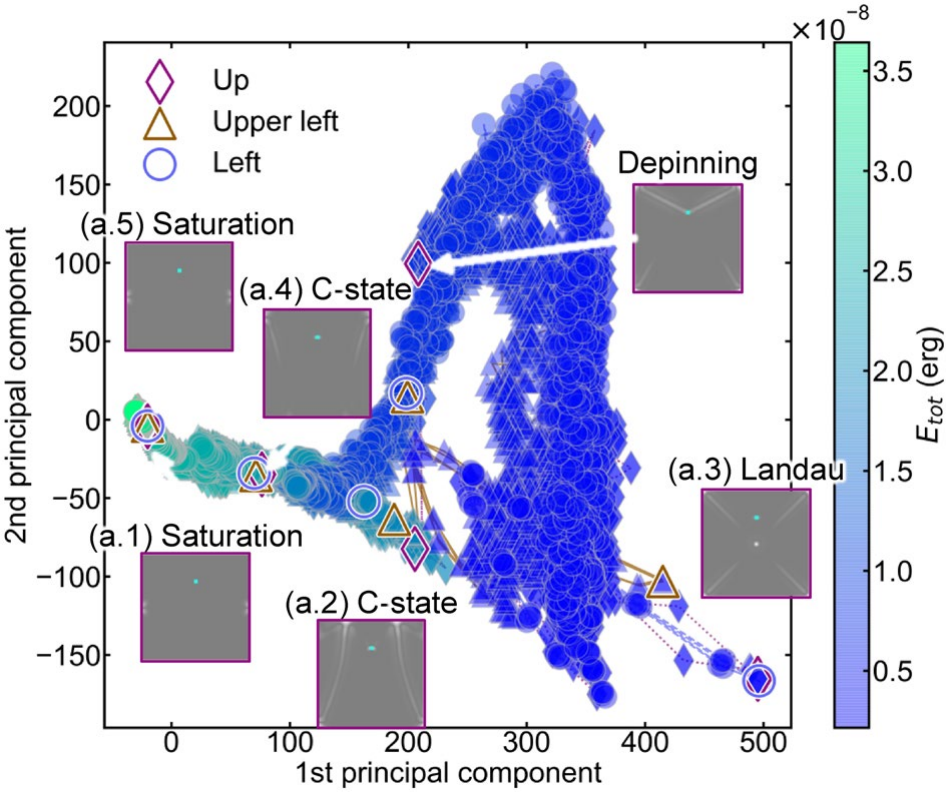
Energy landscape of the magnetization reversal process obtained by PCA and the correlation between the PC1 and magnetization. (**a**) Scatterplot of the dimensionality reduction results of the PCA colored by the total energy ($E_{tot}$). Triangle, circle, and diamond plots correspond to Up, Upper-left, and Left, respectively. The corresponding magnetic domain images are shown. Distance between data points represents the change in magnetic domains. The position and total energy of the data points change continuously according to the magnetization reversal. Continuous changes correspond to domain wall propagation. Jumps correspond to depinning.

Figure 4 shows the dimensionality reduction results obtained using PCA, which is an extended energy landscape. Figure 4 visualizes the trend of data change in a two-dimensional space. The coordinates of the data points are the eigenvalues of each principal component. Changes in data points correspond to changes in PD, thus corresponding to structural changes in magnetic domain. A larger eigenvalue indicates a larger distance from the origin and a larger change in PD. The total energy (sum of the exchange and demagnetization energies) is marked by the color of the data point. This figure visualizes the relationship between the data point travel distance, magnetic domain change, and total energy change, and allows us to analyze the energy costs of structural change in magnetic domain. We analyzed the trend of the data point changes using the eigenvalues of each principal component. The diamond, triangle, and square markers correspond to the samples with defects at Up, Upper-left, and Left, respectively. The position and total energy of the data points generally exhibit continuous changes throughout the magnetization reversal process. The saturated magnetic domain (1) was placed near the origin of the scatter plot. The magnetic domain is constructed by demagnetization. PC1 increases and moves to the C-state (a.2). In the coercivity region, PC1 showed almost the maximum value with the Landau pattern (a.3). After magnetization reversal, PC1 decreases through the C-state (a.4) and returns to saturation (a.5) at the origin. This tendency was reproduced in the Upper-left and Left samples. Correlation analysis of the PC1 and total energy using Pearson’s correlation coefficient yielded a high negative value of − 0.940. Previous PH analysis results for experimental magnetic domains on YIG thin films confirmed that PC1 could be regressed by the second-order polynomial of magnetization^22^. The RR in Fig. 2 shows that the PD varies linearly with energy. The total energy is the quantity of the square of the magnetization. Thus, a second-order polynomial regression could be a reasonable strategy for expressing PC1. We obtained the following regression function:

2 $$ PC1 = f\;\left( M \right) = - 5.02 \times 10^{7} \;M^{2} - 3.73 \times 10^{22} \;M + 328.73 $$

R^2^ of second-order polynomial regression is 0.963. PC1 can be a useful vector for predicting the total energy and explaining the magnetization reversal process. Numerical formalization and detailed dimensional analysis of features require a more detailed evaluation, which will be conducted in a later study.

The sudden breaking jump after the C-state (a.2) is confirmed after combining PC1 and PC2. It corresponds to the drastic change in the magnetic domain from pinning to depinning, namely, the Barkhausen jump^5,18,20,26^. PC2 showed a continuous increase and decrease to reach the Landau pattern (a.3). This corresponds to a continuous change in the magnetic domain and captures the domain-wall propagation process. Accordingly, the magnetic domain change and the total energy in the magnetization reversal process were successfully described in the feature space.

### Gradient analysis and decomposition of energy landscape

This subsection analyzes the energy barriers using a feature-based differentiation. The energy barrier is the external field required for magnetization reversal^37^. It is defined as the effective magnetic field. In the Barkhausen jump, the magnetic domain changes significantly while overcoming the energy barrier^35^. PCA is a dimensionality reduction technique that preserves the distance (difference) between data points. RR allows for a quantitative energy prediction. Therefore, analyzing the derivatives of the energy landscape is a promising prospect for causal analysis. The effective magnetic field in the classical Landau model is defined as the partial derivative of the total energy by magnetization. However, we transform variables using features PC1 and redefine the gradient of the energy landscape while maintaining mathematical consistency. It is given as follows:

3 $$ H_{eff}^{total} = - \frac{{\partial E_{total} }}{\partial M} = - \frac{{\partial E_{total} \;\left( {PC1} \right)}}{\partial PC1} \cdot \frac{\partial PC1}{{\partial M}} $$

The first term is the energy gradient in the feature space obtained by differentiating the energy landscape in Fig. 4a. The second term is the variable transformation term obtained as a linear function using Eq. (2). In the extended Landau model, simple derivative and variable transformation enable us to analyze total energy changes associated with the magnetic domain change.

**Figure 5:**
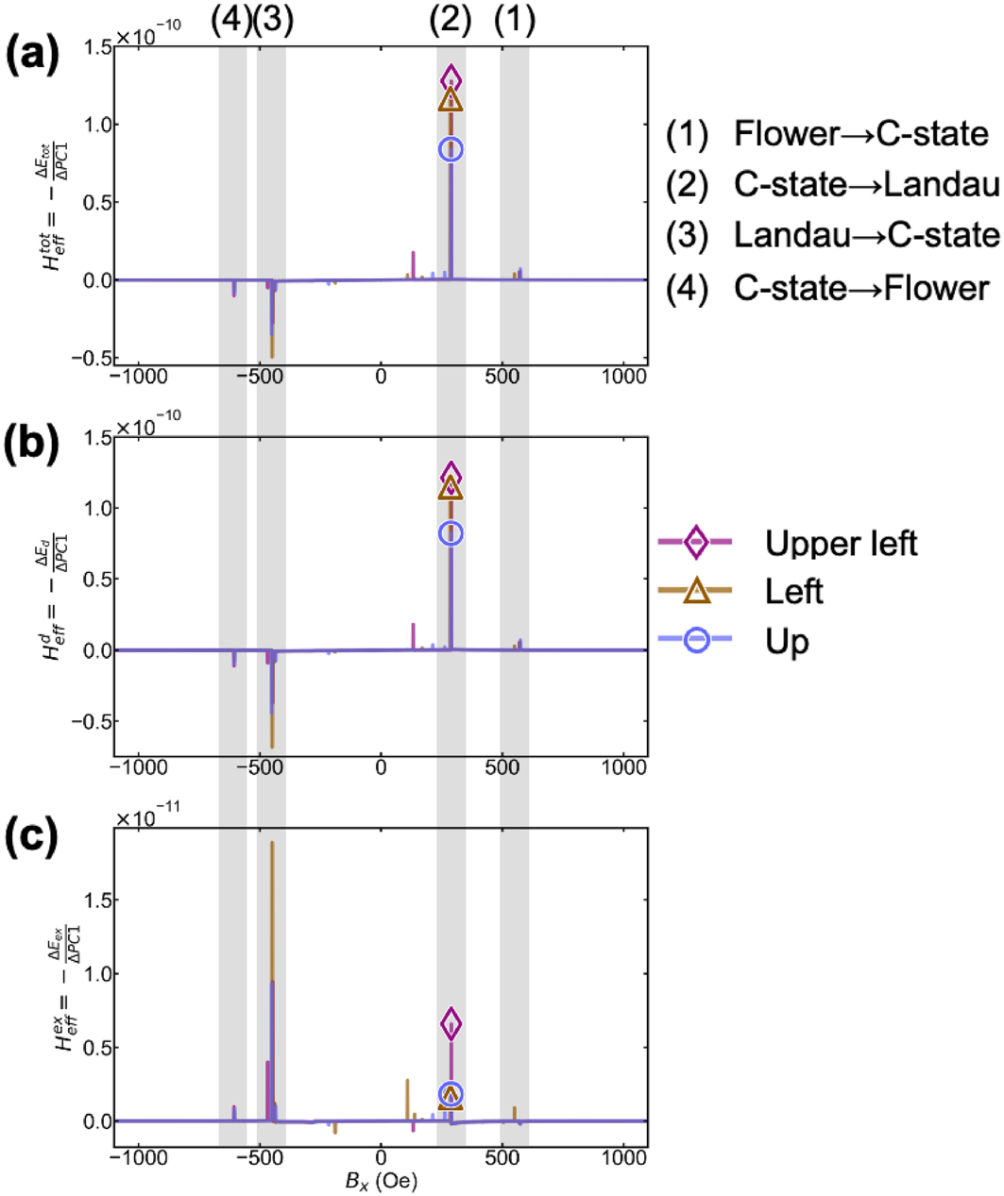
Energy barriers obtained by gradient analysis of the energy landscape of Fig. 4a. (**a**) Energy barrier for total energy. (**b**) Energy barrier for demagnetization energy. (**c**) Energy barrier for exchange energy. The rhombus, triangle, and circle plots are for Up, Upper-left, and Left, respectively. Typical magnetic domain change can be detected as data jump shown as (1) Flower → C-state, (2) C-state → Landau, (3) Landau → C-state, and (4) C-state → Flower.

Figure 5a shows the energy barrier obtained using Eq. (3). The total energy gradient and external field are the vertical and horizontal axes, respectively. A small energy barrier ~ 550 Oe (1) corresponds to the transition from the flower pattern to the C-state during the demagnetization process. By further decreasing the external field, a large energy barrier of approximately 300 Oe (2) was confirmed. It corresponds to the transition from the C-state to Landau pattern. This energy barrier corresponds to pinning. The order of the height in Up, Upper-left, and Left is consistent with the magnitude of the Barkhausen jump in Fig. 2b. Further external field negative reduction reveals an energy barrier at approximately − 400 Oe (3), corresponding to the transition from the Landau pattern to the C-state. This energy barrier is negative. However, the actual magnetization reversal is inhibited since the external field is also negative. A small negative energy barrier at − 600 Oe (4) corresponds to the transition from the C-state to the flower pattern. All energy barriers are obvious and correspond well to the original magnetic domains and magnetization reversal curve, indicating that we could successfully visualize energy barriers in feature spaces.

Next, we linearly decompose the total energy into exchange and demagnetization energies and separately analyze these gradients. Equation (3) is used for each energy term to identify the magnetic effect originating from the energy barrier. Figure 5b,c show derivatives of the demagnetization and exchange energies, respectively. A comparison of the orders is shown in Fig. 5a–c. The demagnetization energy gradient was one order of magnitude larger than that of the exchange energy. This indicated that the demagnetization energy was dominant. This is consistent with the pinning phenomenon observed in soft magnetic materials^38^. The sign of the demagnetization energy barrier is positive when transitioning from (1) the Flower state to C-state and (2) the C-state to Landau state. It is negative when transitioning from (3) Landau to the C-state and (4) the C-state to Flower, as shown in Fig. 5b. The demagnetization energy barrier behavior is similar to the total energy.

The sign of the exchange energy barrier is positive from (1) the Flower state to C-state and (2) the C-state to Landau state, as shown in Fig. 5c. However, the energy barriers were also positive from (3) the Landau state to C-state and (4) the C-state to Flower state. Exchange energy in positive fields acts as an inhibitor of magnetization reversal. However, the sign of the energy barrier is reversed in negative fields, suggesting that it promotes magnetization reversal in the negative field. This means that exchange energy works differently from the demagnetization energy. Identifying such causality is difficult by conventional visual analysis.

We interpreted the sign of each energy term based on magnetic domain formation theory using the LLG equation. In the demagnetization process, demagnetization and exchange energies increase to generate a magnetic vortex during the transition from the (2) C-state to the Landau pattern, which corresponds to pinning. In the transition from (3) the Landau pattern to the C-state, the demagnetization energy maintains the vortex, and exchange energy annihilates it^39^. This is consistent with our results. Therefore, the extended energy model can quantitatively analyze the relationship between magnetic domain change and energy barrier. Additionally, it can explain how each magnetic interaction contributes to the magnetization reversal process. Thus, the cause–effect relationship is successfully analyzed.

### Visualization of causal magnetic effects on the initial magnetic domain

In section “Ridge regression from persistence diagram to demagnetization energy and exchange energy” of “Results and discussion”, we visualized factors contributing to pinning back to the original magnetic domain. To analyze specific energy barriers, we reconsidered the difference in the eigenvalues of PC1 (${\Delta }PC1$). From the PCA definition, ${\Delta }PC1$ was obtained as a scalar by an inner product between ${\Delta }{\varvec{PD}}$ and ${\varvec{u}}_{1}$.

4 $$ {\Delta }PC1 = {\Delta }{\varvec{PD}} \cdot {\varvec{u}}_{1} $$

where ${\Delta }{\varvec{PD}}$ is the difference in the PDs representing the magnetic domain change. ${\varvec{u}}_{1}$ is the principal component vector of the PD with same dimension (${\mathbb{R}}^{d}$) as the PD^30^ (Supplemental Fig. 4). ${\Delta }PC1$ is an explanatory variable of the energy barrier obtained from Eq. (3). It allows bidirectional interconnection between the physical properties and magnetic domain. However, the energy barrier and magnetic domain change are not yet connected because ${\Delta }PC1$ is a scalar, and ${\Delta }{\varvec{PD}}$ is a vector. The analysis of the individual pinning was difficult. It is because ${\Delta }PC1$ and RR coefficients were obtained from the entire magnetization reversal process. The Hadamard product is used to redefine a vector ${\Delta }{\varvec{PC}}1$ preserving the dimension. It also connects the energy barrier with magnetic domain change^31^.

5 $$ {\Delta }{\varvec{PC}}1 = {\Delta }{\varvec{PD}} \odot {\varvec{u}}_{1} \in {\mathbb{R}}^{d} $$

where $\odot$ is the Hadamard product that multiplies every element of ${\Delta }{\varvec{PD}}$ and ${\varvec{u}}_{1}$ to create a vector ${\Delta }{\varvec{PC}}1$, keeping the dimension of original vectors. ${\Delta }{\varvec{PC}}1$ preserves the dimension of the PD and saves the product of ${\Delta }{\varvec{PD}}$ and eigenvector ${\varvec{u}}_{1}$ in each element. We can generate a PD for each energy barrier instead of the entire magnetization reversal. It allows us to focus on a specific pinning. Summing all elements of the ${\Delta }{\varvec{PC}}1$ yields scalar ${\Delta }PC1$.

**Figure 6:**
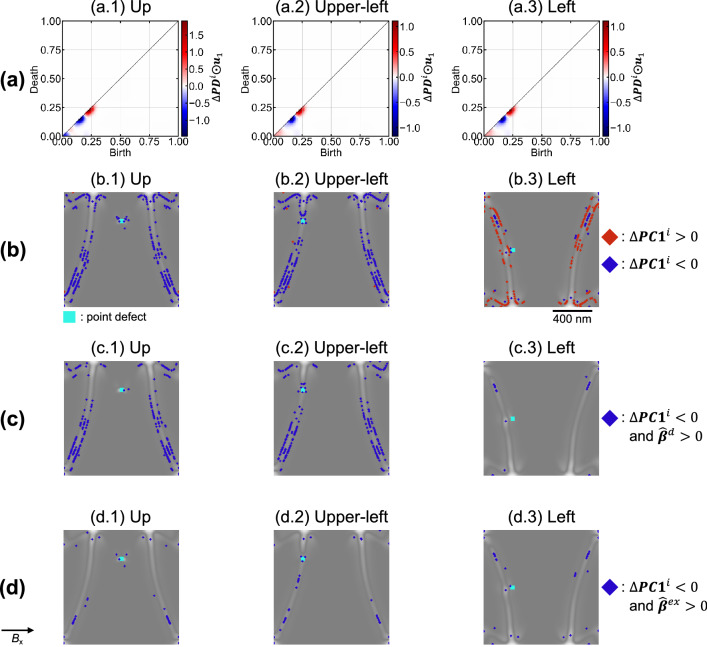
PD reconstructed from the difference of PC1 (${\Delta }{\varvec{PC}}1_{i}$), and visualization on the magnetic domain. (**a**) ${\Delta }{\varvec{PC}}1_{i}$ in depinning produced using Hadamard products. (**a.1**), (**a.2**), and (**a.3**) correspond to Up, Upper-left, and Left, respectively. (**b**) Visualization of total energy contribution ${\Delta }{\varvec{PC}}1_{i}$ to the original magnetic domain. The positive components with ${\Delta }{\varvec{PC}}1_{i} > 0$ and negative components with ${\Delta }{\varvec{PC}}1_{i} < 0$ are red and blue generators, respectively. (**b.1**), (**b.2**), and (**b.3**) correspond to Up, Upper-left, and Left, respectively. In Left, the number of generators for ${\Delta }{\varvec{PC}}1_{i} < 0$ is significantly fewer. It suggests that easier depinning is achieved. (**c**) Visualization of demagnetization energy contribution on the original magnetic domain using the logical sum of ${\varvec{PC}}1_{i} < 0$ and $\hat{\user2{\beta }}^{d} > 0$. (**c.1**), (**c.2**), and (**c.3**) correspond to Up, Upper-left, and Left, respectively. In Left, the number of generators is fewer. The demagnetization energy barrier is significantly smaller. It indicates that the depinning is the easiest. The generators in Left do not appear near defects or at the edge of the dot. This indicates that the magnetic pole divergence and accompanying demagnetization energy loss is adequately suppressed. (**d**) Visualization of the exchange energy contribution to the original magnetic domain using the logical sum of ${\varvec{PC}}1_{i} < 0$ and $\hat{\user2{\beta }}^{ex} > 0$. (**d.1**), (**d.2**), and (**d.3**) correspond to Up, Upper-left, and Left, respectively. The generator detects the center of the domain wall and the defects. The domain wall and defects interact, suggesting that this contributes to system stabilization.

Figure 6a shows the ${\Delta }{\varvec{PC}}1$ produced by the Hadamard product. It focuses on the transition from the (2) C-state to Landau, with the highest total energy barrier. ${\Delta }{\varvec{PC}}1$ is the differential PD between the pinning and depinning. The generator signs in all cases are distinct. This may characterize both the energy difference and the spatial information in the pinning. The PDs in Fig. 3 were produced by the entire reversal process. Therefore, the generator distribution was complex. However, Fig. 6a is produced from pinning only, making the generator distribution simpler. As discussed in section “Ridge regression from persistence diagram to demagnetization energy and exchange energy” at Fig. 3, the negative component of ${\Delta }{\varvec{PC}}1$ (${\Delta }{\varvec{PC}}1_{i} < 0$) contributes a decrease in the denominator, which increases the energy gradient. Thus, it promotes pinning, and inhibits the magnetization reversal. The sublevel of ${\Delta }{\varvec{PC}}1$ is shown in Supplemental Fig. 5.

Figure 6b shows the visualization results of the total energy contribution to the initial magnetic domain using ${\Delta }{\varvec{PC}}1$. The positive ${\Delta }{\varvec{PC}}1_{i} > 0$ and negative ${\Delta }{\varvec{PC}}1_{i} < 0$ components are plotted in red and blue, respectively. At Up (b.1) and Upper-left (b.2), the blue generators are predominant, and red generators are negligible. On the Left (b.3), the red generators are predominant, and the blue generators are almost absent. As discussed in Fig. 3, the generator represents the spatial distribution of the energy barrier. The concentration of the total energy in pinning was successfully visualized in real space. Up (b.1) and Upper-left (b.2) are dominated by the number of negative ${\Delta }{\varvec{PC}}1_{i} < 0$ generators. Whereas the Left (b.3) is dominated by positive ${\Delta }{\varvec{PC}}1_{i} > 0$ generators. Thus, the Left (b.3) is the easiest sample for depinning. Because of the Hadamard product, the energy gradient must be treated with caution. The denominator ${\Delta }PC1$ of the gradient is the sum of each component of ${\Delta }{\varvec{PC}}1$. The positive component qualitatively contributes to an increase ${\Delta }PC1$ while decreasing the energy gradient. Thus, the positive generator detected a decrease in the energy barrier. The negative component of ${\Delta }{\varvec{PC}}1$ (${\Delta }{\varvec{PC}}1_{i} < 0$) decreases the denominator ${\Delta }PC1$. Thus, the negative generator detects an increase in the energy barrier. For the above reasons, the Left sample has the lowest energy barrier and is the easiest to depinning. The energy barriers and the number of negative generators in ${\Delta }{\varvec{PC}}1$ are listed in Table 1. The total energy barrier ($H_{eff}^{tot}$) was the lowest on the Left, and higher on the Upper-left, and highest on the Up specimen. The number of generators was also the lowest in the Left, with higher values in the Upper-left and Left. These results are consistent with each other, suggesting that the feature ${\Delta }{\varvec{PC}}1$ by the Hadamard product and the energy gradient are useful for pinning analysis. The visualized generator can indicate the concentration of total energy in real space.

**Table 1:** List of energy barriers and number of generators in pinning with respect to the defect location. The energies shown are total energy, demagnetization energy, and exchange energy. The number of generators is the number for each energy term.

	Up	Upper left	Left
$H_{eff}^{tot}$	1.28 × 10^−10^	1.16 × 10^−10^	8.39 × 10^−11^
$H_{eff}^{d}$	1.21 × 10^−10^	1.15 × 10^−10^	8.21 × 10^−11^
$H_{eff}^{ex}$	6.59 × 10^−12^	1.60 × 10^−12^	1.82 × 10^−12^
Generator (total)	291	284	43
Generator (demagnetization)	208	199	20
Generator (exchange)	37	35	34

Next, we decomposed the total energy into demagnetization and exchange energies to visualize the originating magnetic effect in the real space. To prepare PDs for each energy component, we focused again on the regression coefficients $\hat{\user2{\beta }}^{d}$ and $\hat{\user2{\beta }}^{ex}$ representing the demagnetization and exchange terms obtained in Fig. 3. $\hat{\user2{\beta }}$ is logically summed with ${\Delta }{\varvec{PC}}1$ representing pinning phenomena. Specifically, we consider the logical sum of the positive element of $\hat{\user2{\beta }}$ and the negative element of ${\Delta }{\varvec{PC}}1_{i}$ to create a PD with information on both energy and pinning.

Figure 6c,d visualize the results of the pinning phenomena for demagnetization and exchange energy, respectively. The defect positions are Up for (c.1) and (d.1), Upper-left for (c.2) and (d.2) and Left for (c.3) and (d.3). The number of generators of demagnetization energy (Fig. 6c.1,c.2,c.3) is 20 for the Left. It is less than 208 for Up and 199 for Upper-left (Table 1). Therefore, the contribution of the demagnetization energy barrier of the left side is significantly smaller than that of the other systems. It indicates that depinning is the easiest. Table 1 shows that the demagnetization energy barrier for the left side is smaller than that for Upper-left and Up. It supports the visualization results. Additionally, the number of generators is reduced in Fig. 3. Only the contribution to pinning is extracted and visualized. The spatial distribution of the generators on the Up shows that there are no generators near the defects or at the edge of the dots. It suggests that the divergence from the magnetic poles and the associated demagnetization energy loss are adequately suppressed.

Next, we discuss the exchange energy visualization (Fig. 6d.1,d.2,d.3). Generators are displayed at the center of the domain wall and near the defects in all samples. Figure 3f visualizes the concentration of the exchange energy in pinning. Focusing on the generators near the defects, the generators in Up (d.1) were clearly distant from the domain wall. Those in Upper-left (d.2) and Left (d.3) were detected inside the domain wall. A previous study reported that the domain walls and defects interact^36^. The exchange energy barrier $H_{eff}^{ex}$ of Up was clearly lower than that of Upper-left and Left (Table 1). Up can be stabilized by approaching the domain wall and defects. It visualizes the spatial distribution of the exchange energy contributing to the stabilization. As discussed in Table 1 and Fig. 5, pinning phenomena in soft magnetic materials are generally dominated by the demagnetization energy. However, this study successfully visualized a small contribution of exchange energy in real space, which has been difficult by conventional analysis.

### Comparison with conventional analysis

Next, we compared it with the traditional energy landscape. Badea et al. analyzed the relationship between magnetic vortex in the Landau state and pinning phenomena for defect-introduced magnetic dots^40^. They analyzed interactions between the pinning potential and defects by developing an energy landscape using a rigid vortex model^40^. Since this model assumes that the magnetic vortex is protected locally, creation and annihilation of the vortex cannot be described. It simplifies the layout between the magnetic domain and defects. Therefore, most spatial information is missing. Due to the strong assumption about the spatial distribution of energy, they cannot treat the energy landscape of the entire reversal process. Magnetization reversal phenomena have also been analyzed based on Neel-Arrhenius and minimum energy paths (MEP). However, constructing the energy landscape of spatially inhomogeneous systems has been difficult^41,42^.

This extended energy analysis could be used for applications as device structure design, such as magnetic random access memory devices. Previous studies have reported the simple defect position dependence of the energy barrier^5,18^. Our results analyze the energy landscape for spatially inhomogeneous systems using topological features. Our results showed that Up has the lowest barrier of the total energy, and the smoothest magnetization reversal was achieved. It suggests that the left side is a suitable device structure for recording information. By performing a complementary analysis of the intermediate coordinates, it could allow optimization of the device structure. It can offer a novel concept for topological inverse design of device structures for energy-saving recording applications in the future.

Conventional LLG simulations and magnetic imaging techniques can provide information on both the magnetic domain structure and hysteresis; however, interpretation of the fine structure and causal analysis is highly difficult. Our proposed model can characterize the microstructure and quantitatively determine the dominant factors of magnetization reversal. It provides a gateway interconnecting the microscopic structure and macroscopic property across the material hierarchy. It will offer significant benefits not only for functional analysis but also for device design.

## Conclusion

We executed causal and bidirectional analysis of pinning phenomena in inhomogeneous magnetic dots. We designed an extended Landau free-energy model using PH, RR, PCA, gradient analysis, and component analysis. We identified that demagnetization energy is the causal magnetic effect underlying the pinning phenomena and visualized them in the initial magnetic domain. A causal and bidirectional analysis interconnecting the microstructure to the macro-property beyond the hierarchy can be conducted. This can open a new possibility to illustrate a serious unresolved issue in spatial inhomogeneity in mesoscale physics.

## Methods

### Micromagnetic simulations

The magnetization reversal phenomenon was calculated using the micromagnetics simulator Mumax3 based on LLG equation^43^. We used the physical parameters of Py (Ni_80_Fe_20_). The saturation magnetization was *M*_s_ = 8.6 × 10^5^ A/m. The exchange stiffness constant was *A*_ex_ = 1.3 × 10^11^ J/m. The crystal magnetic anisotropy constant was *K*_u_ = 0 J/m^44^. The dot was designed as square of dimensions 1.2 μm × 1.2 μm × 80 nm (with a cell size of 4 × 4 × 8 nm). A point defect of 40 × 40 nm was introduced in the Up, Upper-left, and Left (positions are shown in Supplemental Fig. 6). These three configurations represent various nanostructures. The seed value for random number generation was fixed for the three dots. The initial magnetic domain was calculated until the total energy converged satisfactorily. First, an external field was applied from 0 to 5000 Oe in steps of 1 Oe until the saturation state was achieved. Next, a reversal field was applied from 5000 to − 5000 Oe in steps of 1 Oe. The calculations were continued to close one loop of the magnetic hysteresis curve to prepare 20,000 magnetic domain images. The magnetic domain image saves information about the *M*_x_, *M*_y_, and *M*_z_ components. Datasets of demagnetization energy, exchange energy, Zeeman energy, total energy, and magnetization were also prepared.

### Computational persistent homology

We used HomCloud (https://homcloud.dev/index.en.html) developed by Obayashi et al. for PH analysis, machine learning, and visualization^25,45^. Before PH, we took the absolute value of *M*_z_ and normalized its intensity from 0 to 1. In the PH analysis, we performed filtration using the level-set method and output a PD with a 0-dimensional PH. Sublevel-set filtration can extract the features of dark pixels, whereas superlevel-set filtration can extract the features of bright pixels. We obtained a vectorized PD and used it for visualization in real space.

### Machine learning

To perform RR and PCA, the PDs were converted to a persistence image^25^. We set the dispersion in the PD to σ = 0.03; the mesh range to [0, 1]; and the mesh size to 100. We subsequently obtained a 5050-dimensional vector. The sublevel and superlevel of one system were vectorized to obtain a 20,000 × 5050 matrix. The sublevel and superlevel were then combined for three systems to obtain a 60,000 × 5050 matrix. The sublevel and superlevel were standardized and combined to construct a 60,000 × 10,100 matrix for machine learning. In RR, the demagnetization energy and exchange energy were used as the objective variables. Regression was performed using the obtained matrix as the explanatory variable. The regularization parameter *k* was 150. The dimension of unit vector ${\varvec{I}}_{{\varvec{p}}}$ was 10,100 × 10,100. In the PCA, dimensionality of the obtained matrix was reduced up to the second principal component. Machine learning was performed using Python3.6 with the scikit-learn library^46^.

## Supplementary Information

Supplementary Figures.

## Data Availability

The datasets analyzed in the current study are available in the Zenodo repository, 10.5281/zenodo.6570143.
