# Supplementary material for: Causal analysis and visualization of magnetization reversal using feature extended landau free energy
Source: Sci Rep. 2022 Nov 29;12:19892. doi: 10.1038/s41598-022-21971-1 (PMC9709087; doi:10.1038/s41598-022-21971-1)
Supplement: Supplementary file 1 — Supplementary Figures. [file 41598_2022_21971_MOESM1_ESM.docx]

Causal Analysis and Visualization of Magnetization Reversal using Feature Extended Landau Free Energy

Sotaro Kunii^a^*, Ken Masuzawa^a^, Alexandre Lira Fogiatto^a^, Chiharu Mitsumata^a, b^, and Masato Kotsugi^b^

a Faculty of Advanced Engineering, Tokyo University of Science, Tokyo, 125-8585, Japan.

b National Institute for Materials Science (NIMS), Tsukuba, 305-0047, Japan.

*Corresponding author e-mail: kotsugi@rs.tus.ac.jp

# Supplementary figures


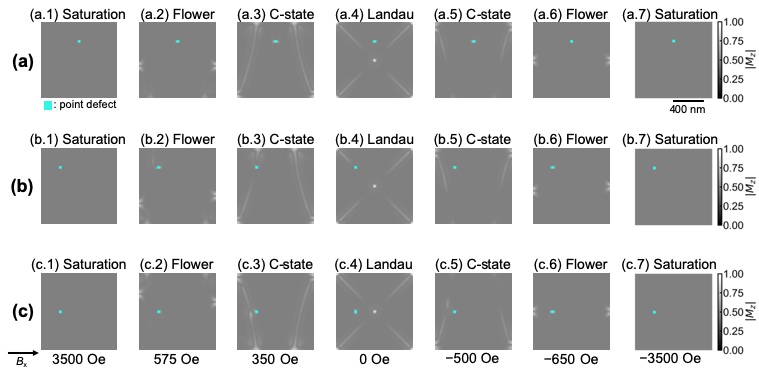


Supplemental Figure 1. Magnetic domain structure images during magnetization reversal process. The light blue points show defects. (a) Magnetic domain image in Up. The magnetic domain structure changes in order of (a.1) Saturation, (a.2) Flower, (a.3) C-state, (a.4) Landau, (a.5) C-state, (a.6) Flower, and (a.7) Saturation during magnetization reversal. (b) Magnetic domain image in Upper-Left. The magnetic domain structure changes in order of (b.1) Saturation, (b.2) Flower, (b.3) C-state, (b.4) Landau, (b.5) C-state, (b.6) Flower, and (b.7) Saturation during the magnetization reversal. (c) Magnetic domain image in Left. The magnetic domain structure changes in order of (c.1) Saturation, (c.2) Flower, (c.3) C-state, (c.4) Landau, (c.5) C-state, (c.6) Flower, and (c.7) Saturation during the magnetization reversal [1].


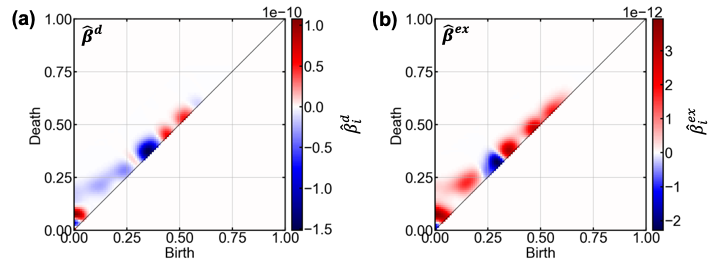


Supplemental Figure 2. *PD*_0_ of sublevel-set in the regression coefficient vector $\hat{\boldsymbol{\beta}}$ obtained by Ridge regression. (a) Regression coefficient vector ${\hat{\boldsymbol{\beta}}}^{d}$ obtained with demagnetization energy as objective variable. (b) Regression coefficient vector ${\hat{\boldsymbol{\beta}}}^{ex}$ obtained with exchange energy as objective variables [3]. The blue-red color scale indicates the weights for X in calculating each energy.


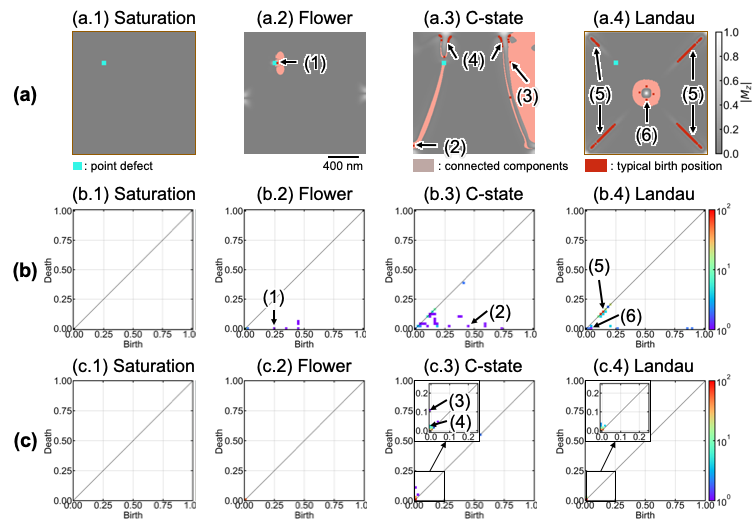


Supplemental Figure 3. Magnetic domain image and zero-dimensional persistence diagram (*PD*_0_). (a) Magnetic domain images during the magnetization reversal process corresponding to (a.1) Saturation, (a.2) Flower, (a.3) C-state, and (a.4) Landau. (b) *PD*_0_ at superlevel-set filtration corresponding to (b.1) Saturation, (b.2) Flower, (b.3) C-state, and (b.4) Landau. (c) *PD*_0_ at sublevel-set filtration corresponding to (c.1) Saturation, (c.2) Flower, (c.3) C-state, and (c.4) Landau. The generators in *PD*_0_ shown as (1) to (6) represent the microstructures of magnetic domains. These generators can visualize the original magnetic domain structure. The center of the island with the corresponding number is visualized in red, and the entire island is in pink. The generator at (birth, death) = (0.251, 0.001) in (1) of (b.2) represents the spatial inhomogeneity of magnetization near the defect in (1) of (a.2), which can be caused by the divergence of demagnetization. The generator at (birth, death) = (0.389, 0.046) in (2) of (b.3) represents to Bloch wall in (2) of (a.3). The generator at (birth, death) = (0, 0.113) in (3) of (c.3) represents to magnetic domain in (3) of (a.3). The generator at death time range in (0.02, 0.03) in (4) of (c.3) represents to end face of domain wall in (4) of (a.3). The generator of birth time range in [0.1, ) lifetime range in [-0.005, ) in (5) of (b.4) represents to fine structure inside the Bloch wall in (5) of (a.4). The generator of birth time range in (0, 0.1) in (b.4) in (6) of (b.4) represents magnetic vortex in (6) of (a.4) [1][2].


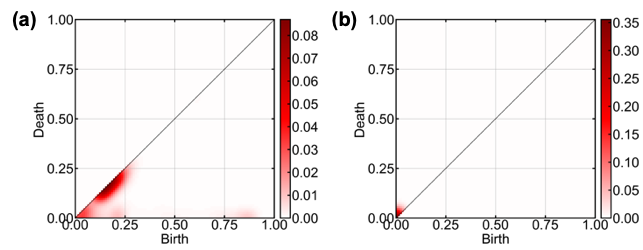


Supplemental Figure 4. $\boldsymbol{u}_{1}$ (eigenvectors of PC1) obtained by PCA. (a) *PD*_0_ of superlevel-set in eigenvectors of PC1; (b) *PD*_0_ of sublevel-set in eigenvectors of PC1 [3]. The color scale indicates the weights of generators in sublevel PD.


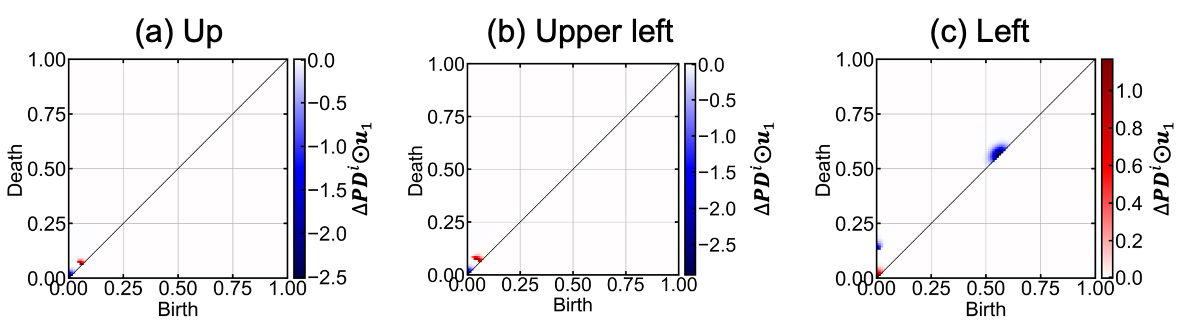


Supplemental Figure 5. $\Delta\boldsymbol{PC}\boldsymbol{1}^{i}$ obtained by the Hadamard product. (a) *PD*_0_ of sublevel-set in Up. (b) *PD*_0_ of sublevel-set in Upper left. (c) *PD*_0_ of sublevel-set in Left [4]. The color scale indicates the weights of generators in sublevel PD.


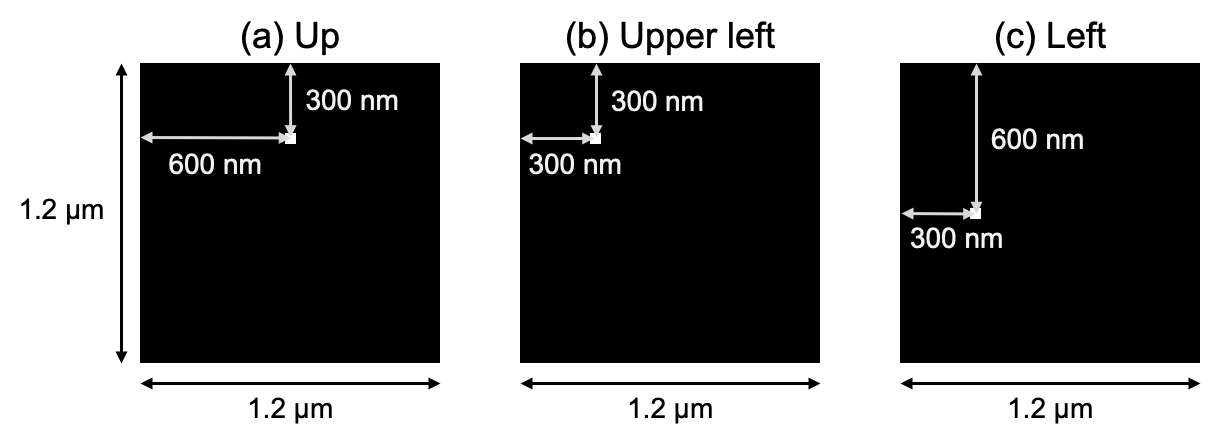


Supplemental Figure 6. Design of the Py magnetic dots. The black area corresponds to Py, and the white region is the defect. (a) Defect configuration of Up. (b) Defect configuration of Upper-Left. (c) Defect configuration of Left.

# References

[1] Rave, W. & Hubert, A. Magnetic ground state of a thin-film element. *IEEE Trans. Magn.* **36**, 3886–3899 (2000). doi:10.1109/20.914337.

[2] Hubert, A. *Magnetic Domains*. *NanoScience and Technology* (Springer Berlin Heidelberg, 1998). doi:10.1007/978-3-540-85054-0.

[3] Hastie, T., Tibshirani, R. & Friedman, J. *The Elements of Statistical Learning*. *Encyclopedia of Systems Biology* (Springer New York, 2009). doi:10.1007/978-0-387-84858-7.

[4] *Matrix Theory and Applications*. vol. 40 (American Mathematical Society, 1990).
